# Development of an Electroactive Hydrogel as a Scaffold for Excitable Tissues

**DOI:** 10.1155/2021/6669504

**Published:** 2021-01-30

**Authors:** Kriti Gupta, Ruchi Patel, Madara Dias, Hina Ishaque, Kristopher White, Ronke Olabisi

**Affiliations:** ^1^Department of Biomedical Engineering, Rutgers University, Piscataway, NJ, USA; ^2^Graduate School of Biomedical Sciences, Rutgers University, Piscataway, NJ, USA; ^3^Department of Biomedical Engineering, The Henry Samueli School of Engineering, University of California—Irvine, Irvine, CA 92697, ZOT Code 2715, USA

## Abstract

For many cells used in tissue engineering applications, the scaffolds upon which they are seeded do not entirely mimic their native environment, particularly in the case of excitable tissues. For instance, muscle cells experience contraction and relaxation driven by the electrical input of an action potential. Electroactive materials can also deform in response to electrical input; however, few such materials are currently suitable as cell scaffolds. We previously described the development of poly(ethyelene glycol) diacrylate-poly(acrylic acid) as an electroactive scaffold. Although the scaffold itself supported cell growth and attachment, the voltage (20 V) required to actuate these scaffolds was cytotoxic. Here, we describe the further development of our hydrogels into scaffolds capable of actuation at voltages (5 V) that were not cytotoxic to seeded cells. This study describes the critical next steps towards the first functional electroactive tissue engineering scaffold.

## 1. Introduction

An important aspect and challenge of tissue engineering is the development of scaffolds that mimic the native environment cells experience. Particularly, challenging is recapitulating the environment of cells originating from excitable tissues such as neural or muscle tissues. For instance, both muscle and neural tissues experience electrical activity in the form of action potentials, while muscle cells also deform during contraction and relaxation. Ideal scaffolds for cells derived from these excitable tissues would provide electrical activity and/or deform to mimic the environment that the cells experience [1].

Electroactive hydrogels may be able to provide such an environment. There are numerous investigations that describe hydrogels that actuate in response to a variety of stimuli. For instance, there are polyelectrolyte-based hydrogels that actuate in response to pH [2–11]; polymers that move in response to temperature [12–18]; polymers sensitive to solvents [6, 8, 19, 20], enzymes [21], or light [22–26]; and electroactive polymers that deform in response to electric signal [27–30]. In all of these cases, applying and removing stimuli resulted in deformation and relaxation of a hydrogel; however, in none of these cases were the hydrogels used as a cell scaffold.

Because excitable tissues such as muscle exhibit electrical activity as well as contraction, our approach to developing actuating cell scaffolds has been to develop a scaffold that also exhibits mechanical actuation in response to electrical signals, an electroactive scaffold. The electrical source of stimulation could potentially be provided by the body's natural electrical impulses or transdermally with microelectrodes at the surface of the skin [31, 32]. Electroactive hydrogels are three-dimensional structures that contain ionic bonds that respond to electrical stimulation by deforming. As such, they have the potential to provide seeded cells with a shortening and elongating environment similar to that of muscle. These hydrogels can harness electrical impulses to power mechanical movement in ionic aqueous solutions and are typically composed of synthetic or natural polymers [33–36]. Specifically, in the absence of an electric field, cations and anions in an electroactive hydrogel are randomly oriented, while in the presence of an electric field, the hydrogels undergo anisotropic swelling [37]. In short, mobile cations in the media migrate towards the cathode, penetrating the hydrogel network and inducing ionization of hydrogel groups on the cathode side of the hydrogel, which causes the hydrogel to swell on the anode side as the ionized hydrogel groups become hydrated [37, 38].

Despite their potential to mimic muscle's microenvironment, there are few reports describing cell response to electroactive scaffolds; they have been predominately used for soft robotics [27–30]. There have been several studies that successfully developed electroactive hydrogels with a stated long-term goal of developing engineered tissues, e.g., artificial muscles, but the conditions required to actuate these hydrogels have been too harsh for cellular environments [39–42]. In general, these electroactive hydrogels are cytotoxic due to either hydrogel formulation, the intensity of the electrical field or voltage applied, or the salinity of the immersion solution required to effect deformation [40–42].

Poly(ethylene glycol) (PEG) is a highly biocompatible polymer that is often blended with other polymers to increase the desired material's biocompatibility [43]. While PEG hydrogels themselves are not electroactive, they can be combined with electroactive hydrogels to increase their biocompatibility. Poly(acrylic acid) (PAA) hydrogels are such electroactive hydrogels that respond to changes in voltage by deforming. When we combined PEG diacrylate (PEGDA) and PAA, the resulting PEGDA-PAA composite hydrogel was both biocompatible and responsive to electrical stimuli [1]. Unfortunately, the high voltages necessary to actuate it was lethal to cells. Herein, we describe our approach to increase the PEGDA-PAA hydrogel flexibility such that it actuated at noncytotoxic voltages and verified this by evaluating the viability of seeded or encapsulated mesenchymal stem cells (MSCs) following hydrogel actuation. Two approaches were undertaken to promote actuation under lower, noncytotoxic voltages, namely, reducing the stiffness of the electroactive hydrogels by altering the hydrogels' material properties (i.e., increasing polymer molecular weight from 10 to 20 kDa) and increasing the hydrogels' aspect ratios. Seeded hydrogels were able to sustain MSC viability after being actuated at 5 volts.

## 2. Materials and Methods

Unless otherwise noted, all reagents used were obtained from Sigma Aldrich (St. Louis, MI, USA).

### 2.1. PEG-RGDS Synthesis

Arg-Gly-Asp-Ser (RGDS) (Tocris) peptide was conjugated with acrylate-PEG-succinimidyl valerate (ACRL-PEG-SVA) (Laysan Bio, Arab, AL, USA) as previously described [44]. A 1.2 : 1 ratio of RGDS (433 g/mol) to ACRL-PEG-SVA (3000 g/mol) was used in the conjugation process. Lyophilized RGDS peptide was reconstituted in PBS (4 mL) in an amber vial. PEG-SVA was dissolved in PBS (2 mL) and dripped into the RGDS solution. The ACRL-PEG-RGDS solution was vortexed and titrated to pH 8.0 using 0.1 M sodium hydroxide. The vial was filled with argon, vortexed, and placed on an orbital shaker for 4 hours at the largest tilt and highest agitation settings. In the first 4 hours, the pH of the solution was checked every 45 minutes and readjusted to pH 8.0 if necessary. The vial was left on the shaker overnight to fully react. After 12–16 hours, the solution was adjusted back to pH 7.0. The reaction was transferred to a 3500 molecular weight cutoff (MWCO) dialysis membrane which had been previously rinsed with Milli-Q water. The reaction was dialyzed against Milli-Q water (4 L), changing the water 4-5 times over a 24-hour period. The reaction was then frozen, lyophilized, and stored under argon at −20°C until use.

### 2.2. Hydrogel Preparation

To achieve different hydrogel stiffnesses, two different hydrogel solutions were prepared by dissolving 10 kDa (original, stiffer formulation) or 20 kDa (modified, more compliant formulation) PEGDA (6.7% w/v; Laysan Bio) and acrylic acid (5% v/v) in phosphate buffer solution. A photoinitiator solution containing 2,2-dimethoxy-2-phenylacetophenone in 1-vinyl-2-pyrrolidinone (ACE-NVP; 300 mg/mL) was prepared and added to the hydrogel solutions (5% v/v) immediately prior to injection into a rectangular glass mold and 2 min exposure UV radiation (365 nm, 10 mW/cm^2^), as previously described [1]. For hydrogels that would be seeded with cells, RGDS was incorporated into the bulk or conjugated to the hydrogel surface, prepolymers were sterile-filtered, and molds and instruments contacting hydrogels were disinfected with 70% ethanol. A PEG-RGDS solution was prepared with PEG-RGDS (3% w/v), HEPES (0.5 M), eosin Y (1% v/v), and ACE-NVP (1% v/v). For bulk RGDS hydrogels, the PEG-RGDS solution (7 mM) was added to hydrogel prepolymer solutions. To create hydrogels with RGDS conjugated to hydrogel surfaces, the PEG-RGDS solution (50 *μ*L) was pipetted onto one face of a hydrogel containing no bulk RGDS and exposed to collimated white light for one minute to photopolymerize the PEG-RGDS onto hydrogel surfaces. After polymerization, hydrogels were removed from molds, washed in Dulbecco's phosphate buffered saline (PBS), and allowed to swell at 4°C for 24 hours in PBS. Hydrogels without RGDS were stained with green food coloring, while hydrogels with RGDS were not exposed to food coloring. In our previous work, the aspect ratio of the electroactive hydrogels was 20 : 4 [1]. Based on subsequent pilot data where aspect ratios between 1 : 1 and 25 : 1 were examined, aspect ratios between 25 : 1 and 15 : 1 were selected for the study. To generate several aspect ratios, the hydrogels were cut to 1 mm width and the following lengths, 25 mm 22 mm 17 mm, and 15 mm (*n* = 4 for each group), resulting in aspect ratios of 25 : 1, 22 : 1, 17 : 1, and 15 : 1. Hydrogels were placed in 6-well plates and over 48 hours were subjected to a minimum of 5 exchanges of PBS and incubated in PBS at −20°C or for RGDS-conjugated hydrogels, complete culture media in a humidified incubator at 37°C with 5.0% CO_2_. The washes removed excess food coloring and unbound RGDS.

### 2.3. Cell Culture

Complete culture medium was prepared with Gibco minimum essential media-alpha (*α*-MEM) with nucleosides supplemented with 10% Gibco fetal bovine serum and 1% penicillin/streptomycin. Human mesenchymal stem cells (hMSCs; Texas A&M) were cultured with complete culture media and passaged every 2-3 days when cultures appeared 70–80% confluent. Cells were expanded until the total cell count exceeded 1.5 million cells.

### 2.4. Cell Seeding or Encapsulation

To harvest cells in preparation for encapsulation or seeding on a hydrogel, the cells were washed with PBS, trypsinized, diluted with complete culture media, spun in a centrifuge at 300 g for 5 minutes, aspirated, and resuspended in complete culture media at desired concentrations. For cell seeding, cells were concentrated to 50 × 10^4^ cells/mL, and the cell suspension (200 *μ*L) was pipetted onto RGDS containing hydrogels (*n* = 30). Cell-seeded hydrogels were then immersed in media and placed in incubators. Complete cell media was changed every two days. For encapsulated cells (*n* = 9), a 2x concentration hydrogel solution was prepared, and a cell suspension at 10^5^ cells per mL was combined at a 1 : 1 volume ratio to create a hydrogel-cell prepolymer solution. The solution was injected into a mold and then polymerized with UV light for 60 seconds. The polymerized, cell-embedded hydrogels were immediately removed from the mold and immersed in cell media. The media was changed every 20 minutes for the first hour and every hour for the next 4 hours to wash the hydrogels of any excess unpolymerized PEGDA and AA. The cell-encapsulated hydrogels were kept immersed in cell media overnight in a humidified incubator with 5% CO_2_ at 37°C. The media was changed once more before cells in the hydrogels were evaluated for viability at 24 hours.

### 2.5. Electrical Actuation of Hydrogels

**Figure 1:**
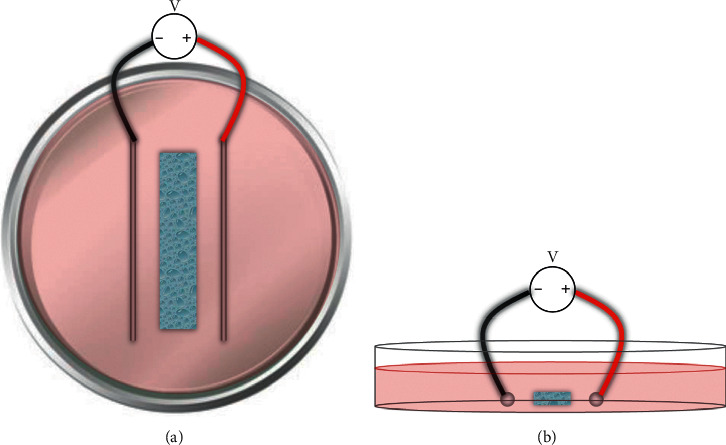
Hydrogel stimulation setup. Two platinum electrodes 3 cm apart were used to apply DC voltage (5, 10, or 20 V) to hydrogels immersed in complete cell media or PBS.

Hydrogels with or without cells were placed in a polystyrene Petri dish and then immersed in complete culture media (for cells) or PBS (for no cells). An Agilent DC power supply (E3646A Agilent Technologies, Santa Clara, CA, USA) was connected to platinum electrodes, which were placed 3 cm apart in the complete culture media on either side of the hydrogel without making direct contact (Figure 1). The hydrogels were then stimulated for 1 min at 5, 10, or 20 V and then placed in fresh media and returned to incubators. Images were taken of green-stained hydrogels before and after stimulation and analyzed with NIH ImageJ to determine the radius of curvature of stimulated hydrogels.

### 2.6. Viability Assays

MSCs encapsulated or seeded on hydrogels were evaluated for viability using an ethidium homodimer-1/calcein acetoxymethyl (AM) LIVE/DEAD® Viability/Cytotoxicity Kit (ThermoFisher, Waltham, MA, USA). For the long-term viability study assessing hydrogel biocompatibility, seeded cells were stained for viability on days 1, 4, 7, 10, 14, and 22. To assess cell survival following hydrogel actuation, viability stains were performed at several timepoints after electrical stimulation: 24 hrs, 48 hrs, and 72 hrs. After each timepoint, fresh dye solution containing calcein AM (2 *μ*M) and ethidium homodimer-1 (4 *μ*M) was prepared. Media was aspirated out, and 1 mL of the dye solution was added to each well (*n* = 4) and incubated for 10 minutes in a humidified incubator with 5% CO_2_ at 37°C before imaging with an epifluorescent microscope (Axio Observer ZI, Zeiss). Cells were observed at 10x magnification with 2 fluorescent channels that labeled cells live (green, ex/em; ∼450/475 nm) and dead (red, ex/em; ∼600/635 nm). Hydrogels were imaged in triplicate.

### 2.7. Statistical Analysis

Pairwise comparisons were conducted using Student's *t*-test to determine statistical significance. For comparisons between more than two groups, a one-factor ANOVA was performed. Statistical significance was set to *p* < 0.05. Post hoc analyses were not conducted as ANOVAs revealed no statistically significant differences. All analyses were performed in Microsoft Excel.

## 3. Results

### 3.1. Electrical Stimulation of Hydrogels

**Figure 2:**
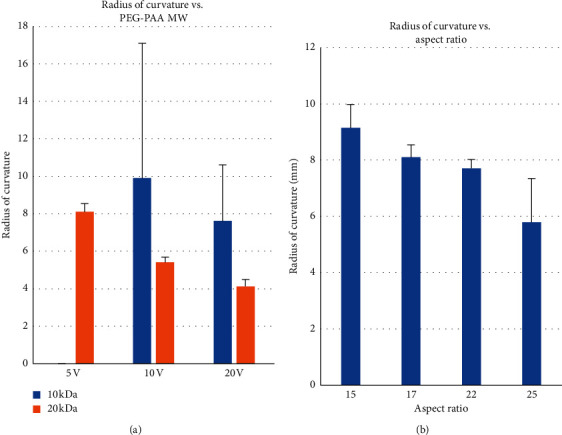
Response of varying hydrogel formulations to electrical stimulation (*n* = 4 per group). (a) Final curvature of 10 kDa and 20 kDa PEGDA-PAA electroactive hydrogels in response to 5, 10, and 20 V stimulation. Smaller radii indicate greater deformation. The 20 kDa PEGDA-PAA molecular weight hydrogels deformed at 5 V to the same extent that the 10 kDa molecular weight hydrogels deformed when stimulated at 20 V. 10 kDa PEGDA-PAA hydrogels did not deform as consistently as 20 kDa hydrogels, leading to high variability. (b) Final aspect ratio achieved by 20 kDa PEGDA-PAA hydrogels. Increasing aspect ratio corresponds to increasing deformation (i.e., decreasing radius of curvature achieved). Error bars show standard error of mean.

**Figure 3:**
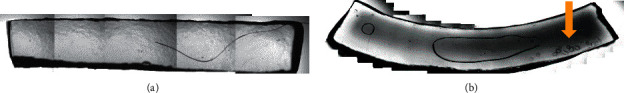
Photomerge of phase contrast images of hydrogel before (a) and after (b) stimulation. Arrow shows accumulation of bubbles on the anode (positive electrode) side of gel at 5x magnification. When electrical stimulation was removed, hydrogels relaxed back into their original conformation. When the polarity of the electrical stimulation was reversed, hydrogels deformed in the opposite direction.

During the 1 minute of 20 V application, all hydrogels deformed. The hydrogels containing 10 kDa PEGDA (i.e., the original hydrogel formulation) deformed slowly and did not achieve their full deformation at the end of the minute. When stimulated for longer than one minute, the hydrogels continued to deform. These hydrogels deformed more slowly at 10 V application and to a lesser extent; they did not deform at 5 V. In contrast, hydrogels containing 20 kDa PEGDA (i.e., the modified formulation) deformed at all levels of stimulation (Figure 2). Hydrogels stimulated in PBS curved slightly more and faster than hydrogels immersed in a complete culture medium. The conjugation of RGDS peptide to the surface of the PEGDA-PAA hydrogels or incorporation into its bulk did not interfere with hydrogel deformation during actuation. Similarly, seeding with cells did not appear to alter the level or rate of hydrogel deformation. When in the presence of an electric field, hydrogels curved towards the cathode (negative electrode) and away from the anode (positive electrode) (Figure 3). Bubbles were visible in the anode side of the hydrogels.

### 3.2. Cell Viability

**Figure 4:**
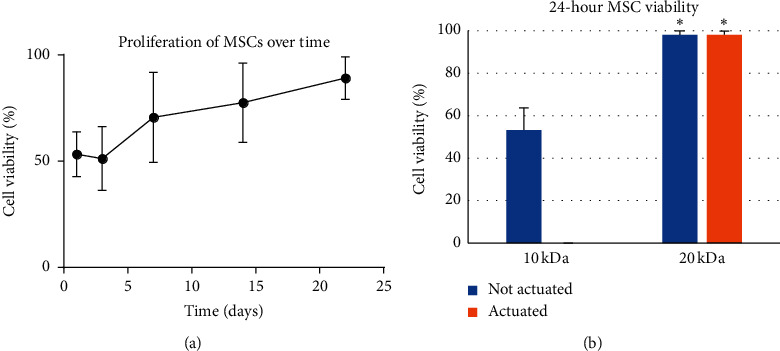
Viability and proliferation of seeded MSCs. (a) Percent viability of MSCs cultured on 10 kDa PEGDA-PAA hydrogels as they proliferated over time. (b) Viability of MSCs after 24 hours of culture, with and without stimulation with the voltage required to effect actuation for 10 kDa (20 V) and 20 kDa (5 V) hydrogels. Note: following stimulation, 10 kDa hydrogels had zero cell viability. Error bars indicate standard deviation (*n* = 9). Asterisks indicate the difference of 20 kDa groups compared to 10 kDa groups.

There was no cell viability in any encapsulated cells. MSCs seeded onto 10 kDa PEGDA-PAA were monitored for viability and proliferation over time and compared to MSCs seeded on 20 kDa PEGDA-PAA (Figure 4). Throughout the 22 days of the viability assessment, the cells proliferated and the percent viability increased.

**Figure 5:**
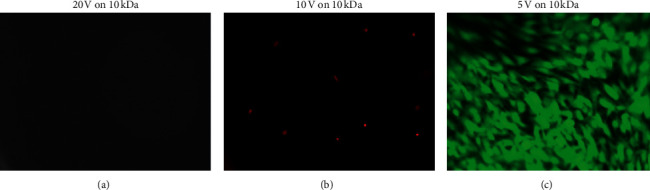
Stained images of cell-seeded hydrogels after actuation. (a) MSCs stimulated at 20 V, (b) 10 V, and (c) 5 V. Cells were stimulated after reaching confluency, stained at 24 hours, and observed at 10x magnification. Stimulation above 5 V appeared to dislodge all cells from hydrogels (A and B). All images were taken with 2 fluorescent channels that labeled cells live (green, ex/em; ∼450/475 nm) and dead (red, ex/em; ∼600/635 nm).

MSCs seeded onto hydrogels and stimulated with 10 V or 20 V were not viable. MSCs on 20 kDa hydrogels showed high viability whether or not they were stimulated at 5 V (Figure 5). Bulk RGDS hydrogels seemed to promote earlier cell attachment, with surface RGDS hydrogels starting with fewer attached cells.

## 4. Discussion

The goal of this investigation was to further develop our electroactive PEGDA-PAA hydrogel for use as a tissue engineering scaffold. Because the hydrogel surface was already biocompatible, but actuation conditions were not, we accomplished this goal by reducing the applied voltage required to achieve hydrogel deformation. By increasing both the PEG polymer molecular weight and the hydrogel aspect ratio, we were able to increase the compliance in our hydrogels. It is well known that increasing the molecular weight of PEG decreases the stiffness of resulting hydrogels [45–48]. In the previous work, we demonstrated that 10 kDa and 20 kDa PEGDA with varying concentrations of PAA range from 60 kPa to 219 kPa [1, 44]. Additionally, according to beam theory, the increased aspect ratio of a beam also increases its compliance. Beam theory also describes the reduced force required to deform a beam when its stiffness is reduced. Therefore, when these PEGDA-PAA hydrogels were subjected to an electric field, it was possible to reduce the field strength to achieve the same deformation with a greatly reduced voltage (from 20 V to 5 V). This, combined with the fact that the immersion solution used is ordinary cell media, makes the stimulation conditions of these hydrogels among the mildest reported in the literature [49, 50]. This may explain why the bulk of research examining electroactive materials do not explore using them as cell scaffolds [50–54].

Although the voltage required to actuate our system is much reduced, at 5 V, it is much larger than physiological voltage levels, which are on the order of mV (e.g., muscle cells depolarize at approximately −70 mV). However, voltage is not as critical as the electric field (*E*=*V*/*d*), which can have very high physiologic values due to small distances. For instance, the electric field of mitochondria is reported to be −3 × 10^7^ V/m (−3 × 10^5^ V/cm) [55]; the electric field of our system is 1.7 V/cm. The optimal external stimulation for engineered cardiac myocytes was determined to be 2.5 V/cm [56]. Engineered muscle constructs were successfully stimulated to achieve 50% of their peak twitch force at electrical field strengths of 1 V/mm (10 V/cm) pulsed for 4 ms [57–59]. Because our PEGDA-PAA polymer can be actuated at electric field strengths below and above these values by altering the voltage or electrode separation, it can be used across a wide variety of applications.

Preliminary results whereby we varied stimulation parameters (e.g., waveform, stimulation voltage, and immersion solution salinity) were not successful in reducing the necessary stimulation voltage. The novelty with respect to our previous work [1] is in the shift to a higher molecular weight and a higher aspect ratio. Although the change is relatively simple and did not rescue encapsulated cells, it did rescue seeded cells, which survived stimulation in this current formulation (20 kDa PEGDA-PAA) but did not survive stimulation with the original formulation (10 kDa PEGDA-PAA). Both 10 kDa and 20 kDa PEGDA-PAA hydrogels proved to be biocompatible scaffolds, but only 20 kDa substrates were capable of supporting cells while actuating. The longitudinal 22-day study demonstrated continued proliferation of MSCs on the surfaces of 10 kDa hydrogels, and the 24-hour comparisons between 10 kDa and 20 kDa polymers demonstrated higher viability on the 20 kDa hydrogels. The 20 kDa poststimulation viability analysis showed high survival and no decrease in viability compared unstimulated controls, demonstrating that stimulation at 5 V was nonlethal to cells and was simultaneously capable of achieving hydrogel deformation. This, combined with the fact that the immersion solution used is ordinary cell media, makes these hydrogels the first PEGDA-PAA electroactive substrates capable of supporting cells during electrical stimulation.

When hydrogels were stimulated, they curved in one direction due to the anisotropic hydrogel swelling caused by the influx of water molecules towards the ionized hydrogel groups on the anode side [37, 38], captured as bubbling in Figure 3. From beam theory, the resulting curve of the hydrogel puts the concave side of the hydrogel in compression and the convex side in tension. This curve could be reversed by reversing the polarity of the electrical field. The greater the hydrogel aspect ratio, the greater the hydrogel curved. Therefore, hydrogel aspect ratio could be used as a variable to subject seeded cells to varying levels of compressive and tensile strains. Thus, our electroactive hydrogel may serve as an important new tool to probe cell response to electrical-mechanical stimulation.

The work herein describes the development of an electroactive material capable of supporting seeded cells and capable of deforming in response to noncytotoxic voltages. One limitation of the hydrogel is that MSCs survived seeding on hydrogel surfaces and stimulation with 5 V but did not survive encapsulation or stimulation at 10 V or 20 V. Encapsulated cell death is likely due to the high concentration of liquid acrylic acid in the hydrogel rather than the exposure to UV light [60]. UV light has been regularly used to encapsulate cells within hydrogels; therefore, it is unlikely that such exposure caused the cell death observed. For instance, we encapsulated cells using UV light (365 nm, 10 mW/cm^2^) for 1 minute. The same type of UV light (365 nm, 10 mW/cm^2^) was used for 1–5 minutes to encapsulate cells in poly (*N*-isopropylacrylamide-) PEGDA hydrogels, and that study further demonstrated that cells in hydrogels exposed to different UV wavelengths had the same relative survival as cells in hydrogels formed in the absence of UV light [61]. However, once hydrogels have been polymerized and swelled to dislodge free PAA not polymerized into the network, the remaining covalently bound PAA is no longer cytotoxic. Thus, design strategies could be employed to encapsulate cells into our PEGDA-PAA electroactive hydrogel. For instance, cells could be encapsulated into biocompatible PEGDA, which is then sandwiched between preswelled PEGDA-PAA polymers. Alternately, PEGDA-PAA could be formed with PEGDA-PAA polymers that have biodegradable peptides incorporated into their PEG backbone, as we previously demonstrated [62]. This would permit the cells to safely migrate through the PEGDA-PAA polymers. An additional limitation of the study is that only a single application at 1 minute was evaluated. Longer durations and multiple applications of electric fields are needed to further investigate the usefulness of the scaffold for long-term stimulation studies. This polymer has multiple applications as a biocompatible electroactive scaffold and could be used to induce static strain (i.e., a constant electrical field) or cyclic strain (i.e., an electrical field with an alternating polarity). A long-term goal is to further develop these hydrogels as a scaffold for muscle tissue engineering that can mimic the muscle environment and is also capable of providing deformation to a variety of cell types. Our hydrogel deforms in response to electrical stimulation when submerged in cell media, a step forward from previous electroactive polymers that require concentrated sodium chloride solutions incapable of supporting cell viability [49]. Thus, this electroactive polymer may be the first step towards developing a muscle scaffold capable of deforming prior to contraction by seeded cells. In essence, our PEGDA-PAA hydrogels could provide tension and compression that the native muscle environment might provide to immature muscle cells. Thus, this hydrogel may lead to the first muscle scaffold that truly mimics the native tissue environment.

## Data Availability

The data used to support the findings of this study are available from the corresponding author upon request.

## Abbreviations

*α*-MEM: Alpha-minimum essential media
AA: Acrylic acid
ACE: 2,2-Dimethoxy-2-phenylacetophenone
ACRL: Acryloyl
GTA: Glutaraldehyde
ECM: Extracellular matrix
HBS: HEPES buffered saline
NVP: 1-Vinyl-2-pyrrolidinone
MSCs: Mesenchymal stem cells
PAA: Poly(acrylic acid)
PBS: Phosphate buffered saline
PCL: Polycaprolactone
PEGDA: Poly(ethylene glycol) diacrylate
PEGDA-PAA: Poly(ethylene glycol) diacrylate-poly(acrylic acid)
SVA: Succinimidyl valerate
UV: Ultraviolet.
